# Post-Orthodontic Relapse Prevention through Administration of a Novel Synthetic Carbonated Hydroxyapatite–Chitosan Hydrogel Derived from Blood Cockle Shell (*Anadara granosa* L.)

**DOI:** 10.3390/dj12010018

**Published:** 2024-01-19

**Authors:** Aanisah Fauziyyah Nurul Hadi, Sabrina Noor Aghniya, Gayuh Abi Haidar, Windy Sepry Marcelina Sihombing, Angelina Sutedjo, Ananto Ali Alhasyimi

**Affiliations:** 1 Undergraduate Program, Faculty of Dentistry, Gadjah Mada University, Sleman, Yogyakarta 55281, Indonesia; aanisah.fauziyyah2902@mail.ugm.ac.id (A.F.N.H.); sabrina.noor.aghniya@mail.ugm.ac.id (S.N.A.); gayuh.abi.haidar@mail.ugm.ac.id (G.A.H.); windy.sepry2903@mail.ugm.ac.id (W.S.M.S.); angelina.sutedjo@mail.ugm.ac.id (A.S.); 2 Department of Orthodontic, Faculty of Dentistry, Gadjah Mada University, Sleman, Yogyakarta 55281, Indonesia

**Keywords:** orthodontic relapse, hydrogel carbonated hydroxyapatite–chitosan, blood cockle shells

## Abstract

Relapse during passive orthodontic treatment is a major issue, with 70–90% frequency. This study examines whether blood cockle shells may be used to extract carbonated hydroxyapatite (CHA)-chitosan (CS). This study also aims to analyze the effect of CHA-CS on orthodontic relapse in rats. This study utilized 18 male Wistar rats which were randomly divided into two groups: CHA-CS and the control group (CG). The rats were subjected to a 35 cN orthodontic force for a duration of 7 days, after which the rats were conditioned to be passive. During this phase, the CHA-CS group received daily administration of CHA-CS hydrogel derived from the blood cockle shell. Subsequently, the appliances were detached to facilitate relapse. The distance between the mesial tips was measured using a digital caliper at three consecutive time points: 1, 5, and 7 days after debonding. The number of osteoblasts, osteoclasts, and fibroblasts was examined using hematoxylin–eosin staining. The data were subjected to statistical analysis using a *t*-test. The relapse distance of the CHA-CS group was lower than that of the control groups on day 7. Histological examinations using hematoxylin–eosin (HE) staining showed a significant increase in osteoblasts, a decrease in osteoclasts, and an increase in fibroblasts during orthodontic relapse movement (*p* < 0.05). This study found that blood cockle shell-derived CHA-CS may reduce orthodontic relapse by increasing osteoblasts and fibroblasts and by reducing the osteoclast number in rats.

## 1. Introduction

Contemporary society is currently experiencing a growing fascination with cosmetic dentistry, and thus, orthodontics is becoming an essential requirement. Orthodontic therapy has emerged as a highly sought-after practice in the field of cosmetic dentistry [1]. Orthodontic treatment is a procedure that utilizes the teeth to achieve straightness, with the dual objectives of enhancing the aesthetic appeal of the dentofacial region and ensuring correct functional alignment [2]. Orthodontic relapse, a frequent challenge encountered in orthodontic therapy, often arises because of the extended and unstable nature of bone remodeling [3,4]. Relapse is the occurrence of the corrected tooth arrangement reverting back to its original pre-treatment position after undergoing treatment [5]. The rate of relapse after orthodontic treatment varies between approximately 70% and 90%, and this issue remains unresolved in the field of orthodontics [6]. The stage of retention is considered to be the final and crucial step in maintaining the proper positioning of dental components following active orthodontic tooth movement. However, there is still a chance of relapse even a decade after the removal of the retainer [7].

Franzen et al. observed that alveolar bone remodeling is a significant factor in the occurrence of orthodontic relapse, according to their animal study [8]. Osteoclasts, osteoblasts, and fibroblasts are the key cellular components that regulate the dynamic process of bone remodeling. A crucial determinant in preventing relapse is the turnover rate of periodontal collagen fibers, which is believed to be controlled by fibroblasts. Osteoclasts disintegrate existing bone, while osteoblasts generate new bone. All of the aforementioned cells cooperate and interact to accomplish bone remodeling [9]. Biological treatments that hinder bone resorption and promote bone formation can effectively reduce the occurrence of relapse. The results suggest that managing the remodeling of alveolar bone following orthodontic tooth movement is an essential approach in preventing relapse [10,11]. The incorporation of hydrogel carbonated hydroxyapatite (CHA) in advanced platelet-rich fibrin serves as an excellent biological retainer to minimize orthodontic relapse. The study utilized a minimally invasive approach to administer a material that promotes bone formation and supports bone growth. This strategy is effective in reducing the likelihood of relapse following orthodontic tooth movement [5,10]. However, given the study’s limitations, such as the lack of aPRF stock, which should be used immediately after preparation, and the intricacy of CHA synthesis, the translation of these findings to the clinical environment requires additional research. Alternatively, Asefi et al. employed two biocompatible hydrogels, specifically a 4% *w*/*v* chitosan and a 10% *w*/*v* gelatin, to impede orthodontic tooth movement. Chitosan, being a highly utilized polymer in the field of medicine, was chosen for this purpose [12]. Nevertheless, within the field of orthodontics, their study stands as the sole existing documentation of its application as a means of transportation. The authors state that chitosan gel at physiological pH, due to its pH sensitivity and mucoadhesive property, make it suitable for topical application in the oral cavity [13].

Chitosan is present in the exoskeleton of invertebrates, specifically in clamshells like blood clam or blood cockle (*Anadara granosa* L.) [14]. Blood cockle shell is found around the world, particularly in coastal areas. With a total production of 5200 thousand tons, cockle is one of the most popular shellfish products. Because the volume of shells exceeds that of shellfish by more than 70%, the number of waste shells is projected to be at least 370 thousand tons each year [15]. They are deposited in coastal regions, resulting in potential ecological issues. Cockle shells have been regarded as an inexpensive reservoir of calcium carbonate CaCO_3_. They consist primarily of CaCO_3_, predominantly in the crystalline forms of calcite or aragonite [16]. The utilization of calcium carbonate derived from cockle shells has demonstrated its efficacy and cost-effectiveness as an adsorbent for the absorption of Pb and Cd in aqueous solutions [17]. Currently, researchers in nanotechnology are highly interested in the use of cockle shell-derived calcium carbonate nanoparticles (CSCaCO_3_NPs) for two purposes: as a nanocarrier for delivering various types of drugs, and as a bone scaffold. This is because CSCaCO_3_NPs have several advantageous properties, including biocompatibility, osteoconductivity, pH sensitivity, slow biodegradation, hydrophilicity, and a high level of safety [18].

The utilization of CHA in the field of dentistry is primarily attributed to its resemblance to bone structure and its capacity to release calcium, hence facilitating bone remodeling [19]. According to a prior study, the process of bone remodeling may be influenced by CHA, which has the potential to enhance the differentiation of osteoblasts while simultaneously reducing the activity of osteoclasts. In recent years, there has been significant interest in the utilization of chitosan (CS) for enhancing the process of bone regeneration, owing to its notable osteoconductivity qualities [20,21]. Chitosan also exhibits involvement in the process of osteogenesis and does not elicit allergic reactions. Furthermore, it is worth noting that the utilization of CS in the process of periodontal tissue regeneration is highly advantageous due to its exceptional bioactive, biointegrated, and conducive features, which ultimately lead to favorable tissue responses [22]. Therefore, the gradual extraction of chitosan hydrogel from blood cockle shells can serve as a viable alternative substance for reducing the occurrence of relapse following orthodontic therapy.

The success of orthodontic treatment relies heavily on the efforts made to limit relapse, which is a crucial consideration given the significant number of individuals who utilize orthodontic devices. Given the significant consumption of blood shells, which results in the generation of shell waste, it is worth noting that there exist certain components such as CHA and CS that possess the capacity to mitigate the occurrence of relapses. Hence, it is imperative to enhance the utilization of calcium hydroxyapatite–chitosan composites derived from blood cockle shells in the context of orthodontic relapse. The objective of this study is to investigate the feasibility of extracting CHA-CS from blood cockle shells as a potential therapeutic agent for reducing relapse following orthodontic tooth movement.

## 2. Materials and Methods

### 2.1. Ethical Approval

The Ethics Committee of the Faculty of Dentistry and Prof. Soedomo Dental Hospital, Universitas Gadjah Mada, approved this study on 14 July 2023, with letter number 131/UNI/KEP/FKG-RSGM/EC/2023.

### 2.2. Synthesis of CHA-CS from Blood Cockle Shells

Blood cockle shell powder was calcined at 800 °C for 5 h in a furnace to convert calcium carbonate (CaCO_3_) into calcium oxide. After mixing with (NH_4_)_2_HPO_4_ and deionized water in a hydrothermal vessel, the calcined powder was baked at 180 °C for 20 h. Hydroxyapatite products were washed with aquades and dried. The hydroxyapatite powder was heated to 800 °C for 1 h, 900 °C for 8 h, and 1000 °C for 16 h. This thermal treatment was conducted in a dry, CO_2_-rich atmosphere. The extraction and conversion of chitin into chitosan are two steps in the production process. Chitin isolation involves deproteination, demineralization, and depigmentation. Cockle shell powder was blended at a 1:10 ratio with 3.5% NaOH for deproteination. This mixture was magnetically stirred for 2 h at 70 °C. The mixture was rinsed with aquades and baked at 60 °C. Demineralization uses 1:10 (weight/volume) deproteination powder and hydrochloric acid (HCl). It was heated to 30 °C and agitated at 75 °C for 1 h. The mixture was filtered, neutralized, and baked at 60 °C. In conclusion, depigmentation uses 1:10 (*w*/*v*) demineralized powder and hydrogen peroxide (H_2_O_2_). This mixture was stirred for 1 h at 50 °C. Next, the mixture was filtered, neutralized, and dried at 90 °C for 2 h. To make chitosan (CS), the chitin powder was deacetylated with a 50% NaOH solution at a weight-to-volume ratio of 1:10. The mixture was heated for 2 h at 95 °C before chilling. The mixture was then filtered, neutralized, and heated in a 90 °C oven again.

### 2.3. Production of CHA-CS Hydrogel

The gelatin solution type B and CS were combined at a ratio of 80:20. The gelatin–CS combination was gradually dissolved in a solution of acetic acid (1% *v*/*v*) and agitated for a duration of 1 h at a temperature of 60 °C until achieving a state of homogeneity. A quantity of 5 g of CHA powder was introduced into the beaker and agitated for a duration of 1 h. A solution of phosphoric acid (H_3_PO_4_) was added to 50 cc of an aqueous medium, and then, it was gradually introduced into the gelatin mixture while being continuously agitated until achieving homogeneity, a process that took around 2 h. The pH of the solution was measured and controlled to maintain an acidity level of 6. The specimens were thereafter subjected to a freezing process lasting 48 h at a temperature of −18 °C, followed by storage within a range of 1.7–3.3 °C.

### 2.4. Fourier Transform Infrared Spectroscopy (FTIR) Analysis

The FTIR technique was employed to investigate the features of functional groups. The FTIR analysis was conducted utilizing a transmission-type FTIR instrument, with a resolution set at 4 cm^−1^ and a total of 32 scans performed. The CHA and CS powders were combined with KBr at a ratio of 1:100.

### 2.5. Evaluation of the CHA-CS Hydrogel

Evaluation of the hydrogel included several tests to determine the feasibility of topical application preparations on the oral mucosa.

*a.*  *pH test*

pH testing was carried out the day before the hydrogel was applied to the oral cavity of Wistar rats. This test used OneMedTM pH-indicator strips of universal pH 0–14 paper. Preparations that met the optimization of the oral cavity interval ranged from 5.5 to 7.5.

*b.*  *Dispersion test*

The hydrogel weighing 0.5 g was placed in the center of the scale paper and covered with mica, added with 50 g and 100 g of ballast, and then observed for 1 min, until a constant diameter was obtained.

*c.*  *Contact angle test*

Contact angle testing was performed using drip pipettes to drip the hydrogel onto hydrophobic surfaces such as glass plates or mica paper. The camera was directed as parallel as possible to the droplet surface, and then, the angle θ was measured between the CHA-CS hydrogel droplets and the hydrophobic surface.

*d.*  *Organoleptic test*

Organoleptic tests are performed using the five senses including color, smell, taste, consistency, and precipitate. The organoleptic test was carried out by 3 respondents after storage of the preparation for 2 days at room temperature and cold temperature around 1.7–3.3 °C.

### 2.6. Animal and Experimental Procedures

The research methodology employed in this study consisted of in vivo experimental laboratory research, wherein control groups and treatment groups of Wistar rats were utilized. The study conducted by Elkattan et al. involved a four-week period during which various observations were made [23]. These observations were categorized into four distinct phases: (1) the initial week, referred to as the acclimatization phase; (2) the second week, known as the insertion and activation phase or the installation phase of the orthodontic appliance; (3) the third week, designated as the stabilization phase, which involved the topical application of hydrogel; and (4) the final week, referred to as the relapse phase. The observations will be conducted during the fourth week following the onset of the relapse phase.

The study animals were 18 healthy male adult Wistar rats (*Rattus norvegicus*) weighing 200–250 g and aged 2.5–3 months. The animals were housed in polycarbonate cages and given a normal pellet diet with ad libitum tap water. Standard climatic conditions were maintained, including 12-h light–dark cycles, a temperature of 21 °C, and a humidity level of 50%. The animals were separated equally into control and treatment groups (each group included 9 animals) and then randomly divided into three subgroups, each with three animals, corresponding to three observation periods, i.e., day 1, 5, and 7 after the appliances were debonded.

The orthodontic appliances were applied to all of the animals. During orthodontic appliance installation, the rats were sedated intramuscularly with 10 mg/kg BW of ketamine (Kepro™, the Netherlands) and xylazine (Xyla™, the Netherlands) mixed at a 1:1 ratio. To shift the teeth distally, all rat upper incisors were given a three-spin loop spring (diameter 2 mm, length 6 mm, and soldered to the orthodontic band) made of 0.012″ stainless steel archwire (DiynaFlex, MO, USA). The appliances were activated by bending the loop until it reached a standard force of 35 g, as measured using a tension gauge (Medkraft Orthodontics, USA). This activation was maintained for a period of 5 days, followed by a stabilization period of 5 days without further reactivation.

During the retention period, a volume of 0.1 mL of hydrogel CHA-CS was administered topically daily into the mesial side of the incisor gingival sulcus. After a 5-day interval, the orthodontic appliance was removed, and the teeth were left without any support to allow the bottom incisors to go back to their original position.

### 2.7. Determination of Tooth Displacement (Distance of Relapse)

The distance between the mesial points of the upper incisors was used to measure the amount of relapse. This measurement was taken on day 1, 5, and 7 after uninstalling the appliances using a 0.01 mm digital caliper (Pro-Max^®^, China). The distance was recorded twice, shortly following debonding (T0) and on the day of sacrifice (T1), and the relapse distance was computed (T2 = T0 − T1). Each measurement was performed by two experienced researchers who were blinded to the given regimen and replicated thrice. The examiners exhibited a strong level of agreement in their analysis (κ = 0.89), suggesting satisfactory intra-examiner and inter-examiner reliability. The average of these values was utilized as a corresponding value for each distance.

### 2.8. Histological Preparation and Analysis

Following debonding, all rats were euthanized at specific time points (after 1, 5, and 7 days) using an overdose of anesthetic, followed by decapitation. The maxillary bones were then carefully dissected and preserved in 10% formaldehyde at a temperature of 4 °C for a duration of 24 h. A 30-day 14% EDTA (Sigma-Aldrich, USA) decalcification was also performed at pH 7.4. The material was dehydrated, cleaned, and immersed in paraffin wax for a period of 12–16 h and then cut into sections in the mesiodistal direction longitudinally, using a microtome blade manufactured by Leica (model 819) in Germany. More precisely, serial sections with a thickness of 5 μm were cut at intervals of 50 μm.

Hematoxylin–eosin staining was utilized for histological observations of osteoblasts, osteoclasts, and fibroblasts. The deparaffination process involved immersing the paraffin blocks in xylol, with each block being treated twice for a duration of 3 min each. The process of rehydration was carried out by employing water in conjunction with a succession of graded alcohols. The samples were subjected to hematoxylin staining for a duration of 6–7 min, followed by washing with running water for 1 min. Subsequently, the samples were subjected to eosin solution staining for a duration of 3 min. Afterward, the samples were rinsed with running water for 1 min, followed by rinsing with 70% alcohol and a final rinse with water for 1 min until the samples were completely clean. Next, the mounting process was carried out using the mounting solution Entellan^®^ and then covered with a cover glass. Ultimately, the desiccated concoction was appropriately marked.

Histological examination was performed on the mesial side of relapse movement, specifically focusing on the pressure side. Five regions of interest (ROIs) were randomly chosen, spanning from the cervical to the apical region of the alveolar bone. The cell count was determined by examining the specimens using a light microscope fitted with a digital camera (OptiLAB LLC Phoenix, USA) at a magnification of 400×. The cell density per field was determined using Image Raster 3.0^®^ (USA). The histological evaluation was conducted by two impartial experts and repeated three times. The examiners demonstrated a high level of agreement in their analysis (κ = 0.88), showing satisfactory reliability both within and across examiners. The osteoclasts, stained with HE, were observed as large, multinucleated cells with pink-stained cytoplasm, found in close proximity to the Howship’s lacuna. The osteoblasts were identified as cuboidal cells with a single, large, deep blue–purple nucleus, and primarily located on the outer surfaces of the alveolar bone. Fibroblasts are typically spindle-shaped cells with an oval flat nucleus. The quantification of fibroblasts was determined through the examination of actively functioning fibroblasts within the periodontal ligament which possess distinct features such as a substantial cytoplasm, chromatin with a smooth texture, and a discernible appearance. The total cell count was derived by computing the average values across five regions of interest (ROIs) from both incisors.

### 2.9. Data Analysis

Parametric tests were utilized for data analysis. Data analysis involved the utilization of the Shapiro–Wilk normality test and Levene’s test of homogeneity, with a significance level established at 95% (*p* > 0.05). An independent *t*-test was used to compare data between the control and treatment groups. The test was performed at a significance level of *p* < 0.05.

## 3. Results

### 3.1. Synthesis of CHA and CS of Blood Cockle Shells

Prior to the synthesis of CHA and chitosan, blood cockle shell powder was subjected to mechanical grinding using a ball mill and subsequently separated using a 100-mesh sieve. From an initial quantity of 2 kg of shells, a yield of 346 g (±35%) of powder with a particle size of 100 mesh was achieved. Subsequently, the synthesis of CHA was conducted using 50 g of powder, resulting in the production of 30 g (±60%) of fully carbonated A-type CHA. Similarly, the synthesis of CS was performed using 50 g of powder, yielding 20 g (±40%) of the desired product.

### 3.2. CHA and CS Fourier Transform Infra-Red (FTIR) Test of Blood Shells

**Figure 1:**
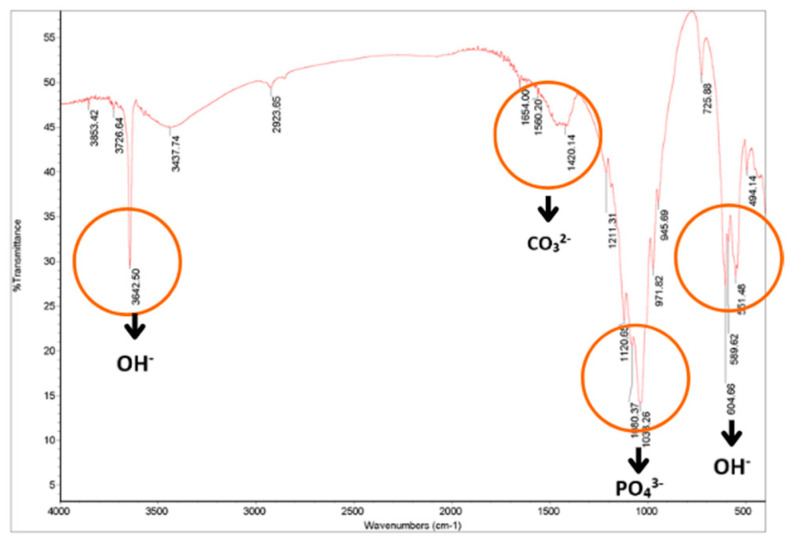
FTIR spectra of specimens tested. The red circles indicate the absorption of waves of blood cockle shell–CHA constituent compounds in the form of OH^−^, CO_3_^2−^, and PO_4_^3−^.

The functional groups of CHA compounds were identified using FTIR spectra. Figure 1 shows the manufactured hydroxyapatite FTIR spectra. OH^−^ groups were detected via absorption peaks at 3642.50 and 604.66 cm^−1^. The OH^−^ hydration group was detected at 3437.74 cm^−1^. The vibrational motion of the CO_3_^2−^ moiety caused modest absorption at 1420.14 cm^−1^. This shows that CO_3_^2−^ ions from the atmosphere have integrated into the outer layer of hydroxyapatite particles. In chemical precipitation CHA synthesis, CO_2_ solutions can cause carbonates to replace hydroxyl or phosphate groups. Absorption at wavenumbers 1080.37 and 1038.26 cm^−1^ suggests PO_4_^3−^ groupings. FTIR research in this work confirms the existence of pure CHA. Table 1 shows the synthesized CHA material’s FTIR results.

**Figure 2:**
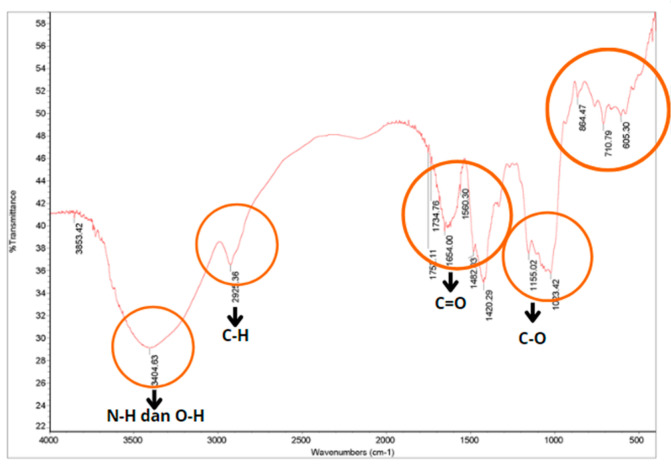
Blood shell chitosan FTIR test results. The red circles indicate the absorption of waves of the compounds that make up chitosan blood cockle shells in the form of N-H, O-H, C-H, C=O, and C-O.

To verify the formation of chitosan from chitin, an analysis of chitosan functional groups is performed using FTIR. Figure 2 shows the FTIR spectrum of synthesized chitosan. From the resulting spectrum, absorption can be seen at wavenumber 3404.63 cm^−1^, which is the result of the OH^−^ group overlapping with N-H absorption. This change in uptake reflects a change from chitin to chitosan. Absorption at wavenumber 2925.36 cm cm^−1^ indicates the presence of a C-H group from the alkane, indicating vibration of the -CH2- group. The transformation from chitin to chitosan can be observed with loss or reduced uptake of C=O (1651.00 cm^−1^) in the chitin FTIR spectrum. There is also a special absorption of chitosan at 1420.29 cm^−1^, indicating the presence of C-N amine bonds. The absorption band at wavenumber 1155.02 29 cm^−1^ is the result of the vibration of the -C-O group. The FTIR results of chitosan synthesis can be seen in Table 2.

### 3.3. Evaluation of Hydrogel Preparations

#### 3.3.1. pH Test

The pH test results show that the pH of the hydrogel is 6 (Table 3). The pH of the hydrogel which ranges from 5.5 to 6.6 shows the best adhesion, as evidenced by the rheological properties with increased compression force that creates deformation of the hydrogel in line with the increase in firmness of the gel due to the increase in viscosity. The high viscosity of the hydrogel strengthens the molecular bonds during the application process (cohesion). The cohesion properties of the hydrogel optimize the distribution of the hydrogel on the oral mucosa and prevent flushing by saliva [26].

#### 3.3.2. Dispersion Test

In this regard, a dispersion test was carried out in which hydrogel preparations were taken in 0.5 mL quantities with a pipette and then dripped onto scale paper to obtain a range of 0.35 mm from the midpoint or a diameter of 0.7 mm.

#### 3.3.3. Contact Angle Test

**Figure 3:**
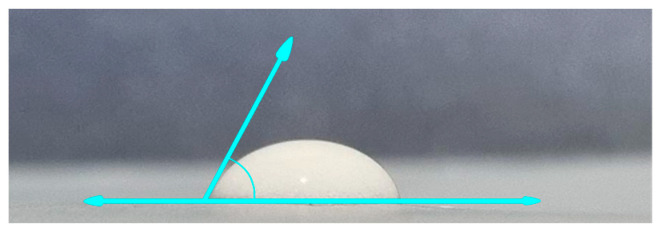
Contact angle test on CHA-CS hydrogel droplets. The blue line indicates the axis of the base surface and the slope of the liquid, forming the angle θ = 72°.

The determination of contact angle testing relies on the demarcation between hydrophilic and hydrophobic regions, denoted by θ = 90° in Figure 3. A lower surface angle signifies a higher degree of hydrophilicity, whereas a larger angle shows greater hydrophobicity. A contact angle of 72 degrees was observed between the hydrogel droplet and the hydrophobic surface, indicating that the angle created was less than 90 degrees. According to a previous study, it can be observed that hydrogel exhibits hydrophilic properties [27].

#### 3.3.4. Organoleptic Test

Organoleptic testing was conducted after a two-day storage period. The result is shown in Table 4. Storage at low temperatures is shown to result in a less intense odor, characterized by a hazy white solution, a sour sweetness, a higher viscosity gel-like texture, and the possibility of precipitation occurring after a standing time exceeding 30 min. The preservation of the substance at room temperature results in the development of a more pronounced odor, a clear white suspension color, a sour and sweet taste, a more liquid gel-like texture, and the possibility of precipitation within a time frame of less than 20 min. The hydrogel’s flavor, color, consistency, and sedimentation rate can be influenced by variations in temperature [28].

### 3.4. Relapse Distance and Histological Analysis Results

**Figure 4:**
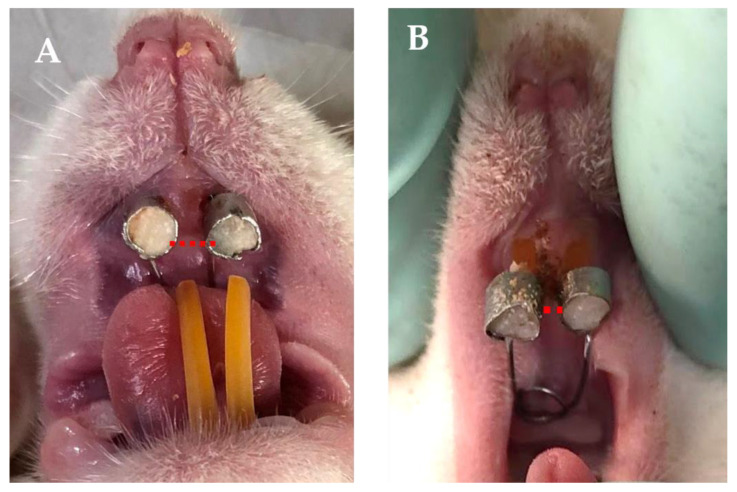
The relapse distances of both groups were measured seven days after debonding. The administration of CHA-CS hydrogel (**A**) effectively reduced relapse in the experimental group (**B**) compared to the control group.

**Figure 5:**
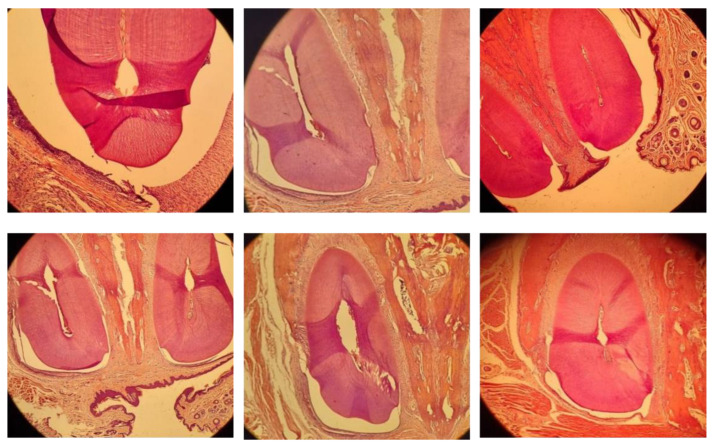
Histology of upper incisors. The upper part of the figure depicts the control group 1, 5, and 7 days after debonding, while the lower part of the figure shows the CHA-CS group at the same time points (400× magnification).

Relapse distance measurements were taken on day 1, 5, and 7 after debonding. Treated animals had a reduced relapse distance over the whole observation period, as compared to the control group (Figure 4). Figure 5 illustrates the contrasting histological appearances between two groups. Table 5 displays the average and standard deviation measurements of the relapse distance, as well as the quantities of osteoclasts, osteoblasts, and fibroblasts in both the control and treatment groups (using CHA-CS hydrogel). A significant difference was noted in the quantity of osteoclasts; the treatment group exhibited a decrease in comparison to the control group on day five following debonding. On the contrary, the number of osteoblasts in the CHA-CS group were considerably larger than those in the control group on day seven following debonding. In addition, the quantity of fibroblasts was substantially greater on day 1, 5, and 7 in the CHA-CS group compared to the control group after orthodontic relapse (*p* < 0.05).

## 4. Discussion

Tissue engineering technologies have been advocated for the manipulation of alveolar bone remodeling, the prevention of orthodontic relapse, and the enhancement of tooth position stability. Due to its precise regulation of calcium release and ability to produce bone, carbonate apatite (CHA) holds significant promise for bone tissue engineering [29]. The findings indicated a notable increase in osteoblasts in the group treated with CHA-CS hydrogel. CHA stimulates bone remodeling by increasing the concentration of calcium and phosphate in the surrounding region, which are crucial for bone formation. Osteoblast activity is regulated by the secretion of calcium and phosphate ions into the adjacent tissue. Elevated concentrations of extracellular calcium impede the formation of osteoclasts while stimulating DNA synthesis and chemotaxis in osteoblastic cells [5,30]. In the interim, it has been observed that CHA induces an upregulation in the expression of transforming growth factor-β1 (TGF-β1) and bone morphogenic protein-2 (BMP-2), both of which play a significant role in the process of osteoblastogenesis. The proliferation of osteoblasts is enhanced by TGF-β1 through the recruitment of osteoblast precursors or matrix-producing osteoblasts, as well as the inhibition of osteoblast death. Transforming growth factor-beta 1 (TGF-β1) exerts a significant impact on the process of bone formation, as it possesses the ability to enhance the proliferation of fibroblasts and accelerate the production of collagen [10].

The results demonstrated a considerable increase in fibroblast levels in the treatment group. Yamamoto et al. initially documented that transcription factors, including Runx2, Osterix, and Oct3/4, L-Myc (RXOL), can be employed to directly transform fibroblasts into osteoblasts [31]. Given the pivotal role of osteoblasts in bone formation, the direct conversion of fibroblasts to osteoblasts could serve as an innovative approach to induce bone regeneration. Osteoblasts and osteoclasts are key components in the regulating mechanism of the relapse process, as they are involved in the processes of bone production and resorption. During the process of relapse, the side of tension that was initially established as a result of orthodontic tooth movement transitions to the side of pressure. This transition is accompanied by an elevation in the differentiation of osteoclasts, leading to the resorption of bone tissue. In contrast, the region of higher pressure that was initially established undergoes a transformation into a region of strain, which in turn promotes the differentiation of osteoblasts, thus initiating the process of bone formation [5,11,12].

The groups who received CHA-CS treatment showed a decreased number of osteoclasts. Regarding the mechanism, CHA improves bone remodeling by elevating calcium and phosphate concentrations in the nearby surroundings. Furthermore, elevated amounts of calcium outside the cells promote the production of DNA and the movement of osteoblastic cells while also preventing the creation of osteoclasts [32]. Previous research has indicated that the rise in phosphate ion levels can inhibit the formation of osteoclasts. This inhibition occurs not only by increasing osteoprotegerin levels but also by directly triggering osteoclast apoptosis [33]. A notable reduction in the number of osteoclasts has been linked to a delay in orthodontic tooth movement [34]. The administration of CHA has the capacity to reduce orthodontic relapse by promoting the expression of osteoprotegerin (OPG) and lowering the amount of receptor activators of nuclear factor-κB ligand (RANKL). The outcomes of this study suggest that local injection of CHA could effectively inhibit osteoclastogenesis and osteoclast activity, leading to potential improvements in orthodontic retention [5]. OPG is an endogenous receptor that is produced by osteoblasts. Its main function is to suppress the formation and function of osteoclasts by binding to the RANKL and preventing its interaction with the receptor activator of nuclear factor kappa-B (RANK). The interaction between RANKL and the RANK receptor triggers the swift transformation of hematopoietic osteoclast precursors into fully developed osteoclasts [35].

Another benefit of CHA is its ability to serve as a drug delivery system in controlled release technology [36]. A current concern in tissue engineering involves the development of precise techniques for delivering bone tissue enhancement. The controlled release system is considered promising due to its ability to transform low molecular weight materials into a higher molecular weight system, hence limiting degradation prior to the onset of medicinal action. To regulate the water content of the hydrogel system, gelatin hydrogel was chosen for this investigation due to its capacity to offer regulated release and degradability using a cross-linking method. Enzymatic degradation of the hydrogel results in the production of water-soluble gelatin fragments, which enables the release of bioactive-loaded components [37].

We encountered a limitation in our research. For the purpose of this study, we opted to utilize HE staining as a means of identifying and quantifying osteoclasts, osteoblasts, and fibroblasts. This cell-counting approach is somewhat subjective. Hence, additional research utilizing distinct biomarkers to ascertain the number of osteoclasts, osteoblasts, and fibroblasts is necessary to enhance the robustness of this work.

## 5. Conclusions

The extraction and synthesis of CHA and CS from blood cockle shells is supported by the presence of identical functional groups, as demonstrated by the results of the FTIR test. The examination of the preparation yielded several findings. The hydrogel exhibited certain features at pH 6, including a contact angle of 72 degrees (indicating hydrophilicity), a dispersion distance of 0.7 cm, and good organoleptic properties when stored at cold temperatures. During a seven-day in vivo experiment investigating the relapse phase, it was noted that the application of CHA-CS hydrogel derived from blood cockle shells successfully decreased relapse after orthodontic tooth movement by significantly enhancing the number of osteoblasts and fibroblasts while simultaneously reducing the number of osteoclasts. Additional research is necessary to determine the impact of varying doses of carbonated hydroxyapatite–chitosan hydrogel, generated from the blood cockle shell, on orthodontic relapse and its effectiveness in humans.

## Figures and Tables

**Table 1:** FTIR CHA spectrum of blood cockle shell.

Types of Bonding	Wavenumber (cm^−1^) of Hydroxyapatite Blood Shell	Wavenumber (cm^−1^) of Hydroxyapatite According to the Literature [24]
OH^−^	3642.50 dan 604.66	3575
OH hydration	3437.74	3423
CO_3_^2−^	1420.14	1470–1420
PO_4_^3−^	1080.37 dan 1038.26	1085–1092

**Table 2:** FTIR spectrum of chitosan blood cockle shell.

Types of Bonding	Wavenumber (cm^−1^) of Chitosan Blood Cockle Shell	Chitosan Wavenumber (cm^−1^) of Hydroxyapatite According to the Literature [25]
N-H bond	3404.63	3500–3100
O-H bond	3404.63	3400–3200
C-H bond	2925.36	3000–2850
C=O bond	1651.00	1654–1541
C-N amine bond	1420.29	1400
C-O bond	1155.02	1200–1180

**Table 3:** Hydrogel pH test.

**Day**	0	1	3
**pH test results**	6	6	6

**Table 4:** Organoleptic test.

Control	Smell	Color	Taste	Consistency	Deposits
**Cold Temperature**	Less concentrated	Cloudy white suspension	Sweet–sour	Solid gel	>30 min
**Room Temperature**	Concentrated	Clear white suspension	Sweet–sour	Liquid gel	<20 min

**Table 5:** Means, standard deviations, and *t*-test results of relapse distance (mm) and osteoblast, osteoclast, and fibroblast count (cells/field) of two groups tested on day 1, 7, and 14 following debonding.

Parameters	CHA-CS Group	Control Group	*p*-Value
**Relapse Distance**			
Day 0	1.13 ± 0.58	0.75 ± 0.05	0.105
Day 5	0.85 ± 0.09	0.77 ± 0.31	0.507
Day 7	0.75 ± 0.05	0.80 ± 0.30	0.790
**Osteoblasts**			
Day 1	24.80 ± 5.59	20.61 ± 1.82	0.179
Day 5	21.34 ± 1.87	17.06 ± 3.41	0.076
Day 7	26.68 ± 3.72	18.14 ± 2.71	0.004 *
**Osteoclasts**			
Day 1	2.80 ± 0.27	2.81 ± 0.45	0.699
Day 5	1.29 ± 0.45	2.31 ± 0.57	0.025 *
Day 7	1.32 ± 0.27	2.22 ± 0.84	0.095
**Fibroblasts**			
Day 1	13.63 ± 3.41	8.21 ± 3.11	0.032 *
Day 5	13.12 ± 2.53	7.20 ± 2.77	0.016 *
Day 7	12.81 ± 2.33	6.20 ± 1.79	0.008 *

* *p*-value < 0.05: significant differences between groups.

## Data Availability

The original contributions presented in the study are included in the article, further inquiries can be directed to the corresponding authors.
